# Gestational exposure to HIV drugs alters intestinal mucosa-associated microbial diversity in adult rat offspring

**DOI:** 10.3389/ebm.2025.10564

**Published:** 2025-08-13

**Authors:** Yaswanthi Yanamadala, Chandra Mohan Reddy Muthumula, Kuppan Gokulan, Kumari Karn, Vicki Sutherland, Helen Cunny, Janine H. Santos, Sangeeta Khare

**Affiliations:** ^1^ Division of Microbiology, National Center for Toxicological Research, U.S. Food and Drug Administration, Jefferson, AR, United States; ^2^ Division of Translational Toxicology, National Institute of Environmental Health Sciences, Research Triangle Park, NC, United States

**Keywords:** antiretroviral therapy (ART), ARV, abacavir, lamivudine, dolutegravir, microbiome, gut mucosa-associated microbes

## Abstract

The antiretroviral (ARV) drug combination of abacavir sulfate, dolutegravir, and lamivudine [ABC/DTG/3TC; Tri combination Anti-retroviral therapy (TC-ART)] has revolutionized HIV treatment by effectively targeting different stages of viral replication. Despite its therapeutic efficiency for maintaining low viremia in the mother during pregnancy, there are concerns for long-term liabilities in offspring that are indirectly exposed during vulnerable periods of development. The commensal microbiota plays a crucial role in maintaining overall gut health, and disruption of the microbiome is often linked to various extraintestinal effects such as immune dysregulation and inflammation. We recently reported the effects of this drug combination in altering fecal microbiome composition of aged rats perinatally exposed to ABC/DTG/3TC-ART. The fecal microbiome can provide only a snapshot of the composition of microbial community at the end of the digestive tract, which may not reflect the microbial population interacting with ileal mucosa. Thus, the current work reports the effects of this drug combination in the gut mucosa-associated microbiome of the same animals, which showed significant microbial diversity and species richness in high dose exposed female adult offspring, along with dose-dependent changes in Firmicutes/Bacteroidetes ratio. The high dose exposure also showed an increase in opportunistic bacterial species in male animals. Overall, we found that, similar to the fecal microbiome, perinatal exposure to TC-ART led to sex- and dose-dependent alterations in the gut mucosa-associated microbial population in aged rats, suggesting that early life exposure to these drugs may influence gut mucosa-associated immune responses and intestinal permeability.

## Impact statement

This study shows that prenatal and lactational exposure to the Anti-retroviral therapy (TC-ART) drug combination (ABC/3TC/DTG) is associated with sex-dependent alterations in the ileal mucosa-associated microbiome composition when offspring are evaluated at 12 months of age. These findings emphasize the importance of including microbiome analyses in studies aiming at evaluating the long-term health effects of TC-ART exposure during early development.

## Introduction

Human immunodeficiency virus (HIV) is a retrovirus that primarily targets the immune system, making it difficult for the body to fight against infections and diseases [1]. Without treatment, HIV can progress to AIDS (acquired immunodeficiency syndrome), which can be fatal [2]. Anti-retroviral (ARV) drugs have revolutionized HIV treatment by suppressing the replication of HIV, preventing it from progressing to AIDS. They have turned HIV from a fatal disease into a manageable chronic condition [2]. One such combination of drugs that has been used is abacavir sulphate, dolutegravir, and lamivudine [3]. Abacavir and lamivudine inhibit HIV reverse transcriptase and cause premature termination of viral DNA synthesis by incorporating non-functional nucleotide analogs into growing DNA chain [3, 4], while also having off target effects on the mitochondria [5, 6]. Millions of individuals globally are on Tri combination Anti-retroviral therapy (TC-ART), including pregnant women, to reduce the risk of mother to child transmission [7–9].

The gut microbiome is important due to its beneficial functions and its involvement in maintaining gastrointestinal homeostasis, health, and disease progression [10]. ARV drugs particularly nucleoside reverse transcriptase inhibitors (NRTIs) (lamivudine), are known to affect host mitochondria by inhibiting mitochondrial DNA polymerase γ, leading to mitochondrial DNA depletion and dysfunction [11, 12]. Considering the evolutionary origin of mitochondria from symbiotic bacteria and similar biochemical pathways between mitochondria and prokaryotes, it is plausible that gut microbiome can be inadvertently affected by ARV drugs. However, most of the anti-retroviral drugs are not investigated for changes in gut bacterial community and overall gut health. Recent studies have shown that exposure to ARV’s can have an impact on the host’s gastrointestinal health including altered microbiome and cytokine profiles [13, 14]. Microbial dysbiosis can have several downstream effects, including changes in immune function, intestinal mucosal permeability, and imbalance in other functional organs [15–17]. For instance, dysbiosis in the ratio of Firmicutes and Bacteroidetes is associated with cardiac diseases [18], while increased *Bacteroides* levels are observed in liver ailments, with the degree of alteration correlating to the severity of the condition [19]. Similarly, decreases in butyrate producing bacteria have been associated with increased immune activation and microbial translocation [20], suggesting a relationship between microbial dysbiosis, and changes in cytokine profiles, and intestinal permeability.

The intestinal barrier is a complex multilayer system with an external physical “mucosal barrier,” epithelial cells, and an internal immunological functional barrier in the form of Payer’s patches [21]. The mucus layer on the top of the gut mucosa hosts several commensal bacteria and serves as a defense line along with the tight epithelial layer. Disruption in the barrier can compromise the integrity of the gut ecosystem, which is associated with several pathological conditions including lung, cardiac, neuronal, and autoimmune diseases [22, 23]. It is widely accepted that newborns are sterile at their birth, and they develop the microbiota through maternal exposure, surrounding environment, and through feeding practices [22]. Interestingly, the gut microbiome also plays a major role in infant immune system development, highlighting the importance of maternal gut health in shaping the offspring health [24, 25]. It is becoming increasingly evident that changes in the gut microbiome influence the development of various diseases in later life [26]. While most of the research is primarily focused on the direct effect of the drug on the host, there is limited information on the effect of the drug on the gut health of the progeny. We recently showed that perinatal exposure to this ARV combination altered the fecal microbiome of aged rats (12 months old) in a sex- and dose-dependent manner [27]. This study expanded those findings by exploring the changes in gut mucosal microbiota of the same animals, providing knowledge on the effects of drug exposure in the intestinal mucosa associated microbiome rather than focusing on the fecal -microbiome at the distal end of the intestinal tract.

## Materials and methods

### Animal housing, care, treatment, euthanasia, and sample collection

**FIGURE 1:**
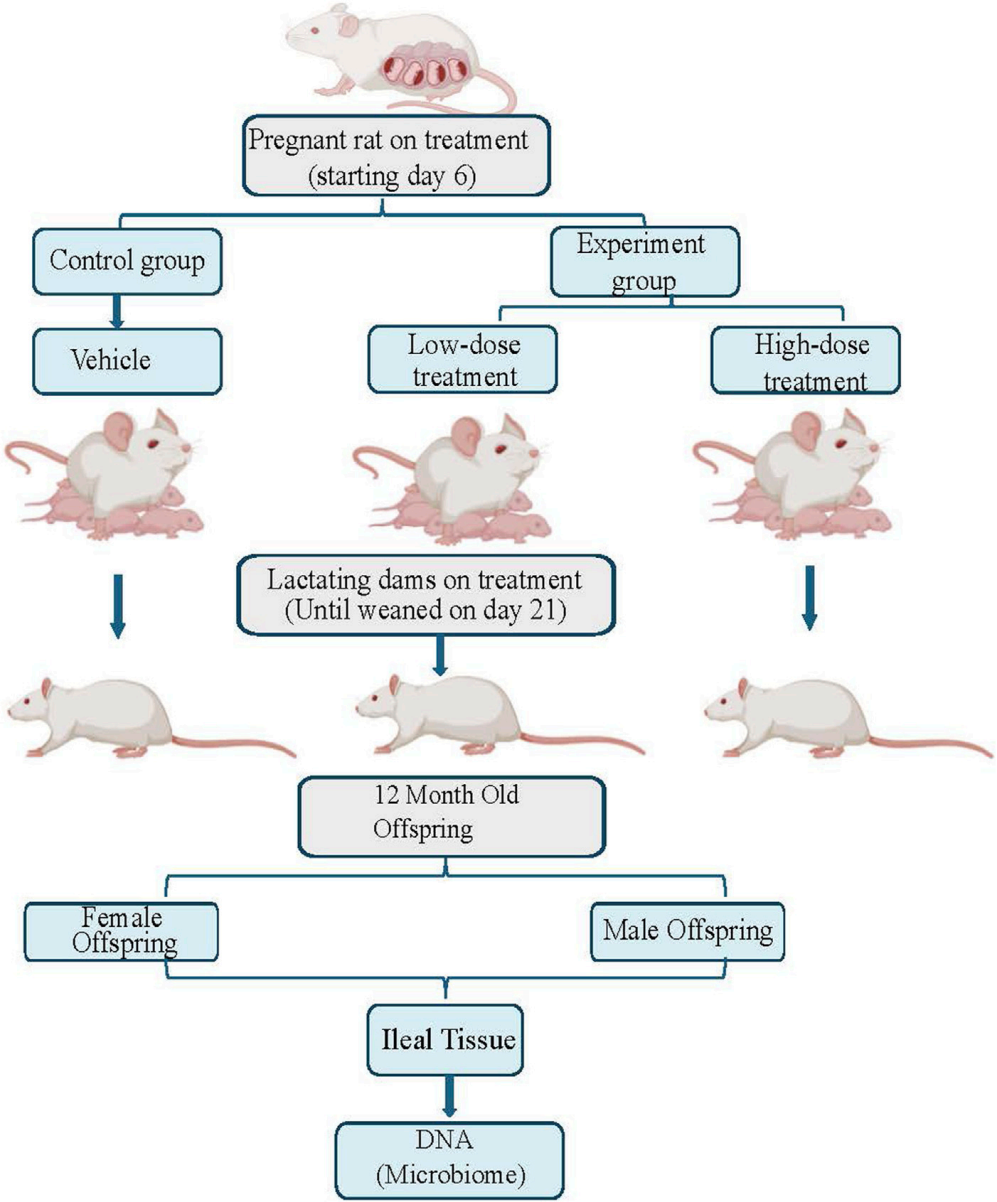
This figure illustrates the experimental flow for this study, which includes three treatment groups: control, low dose, and high dose of tricombo drug treatment. Pregnant rats were treated until the rat pups were weaned on postnatal day 21. DNA, was collected from the ileal tissue of 12-month-old rat offspring for microbiome analysis.

Time-mated Sprague Dawley rats (Hsd:SD) were obtained from Envigo (Indianapolis, IN). All animals (pregnant female rats and their male and female offspring) were housed in the animal facility at AmplifyBio, West Jefferson, OH, an independent, scientific contract research organization. Rats were approximately 11–14 weeks of age upon receipt. The facility’s Institutional Animal Care and Use Committee (IACUC) reviewed the protocol and approved it. The IACUC number for this protocol is T06055. Animals were housed in polycarbonate cages with irradiated hardwood bedding chips (Sani Chips^®^; Envigo, Madison, WI). During gestation and lactation, rats were provided natural crinkled kraft paper for enrichment (Crink-l’nest™, The Andersons; Maumee, Ohio). Offspring remained with their respective dams until postnatal day (PND) 21. After weaning, F1 offspring were provided polycarbonate rectangular shelters (Rat Retreats™, Bio-Serve; Flemington, NJ) as enrichment and were group housed by sex, up to 5 per cage. During gestation and lactation, animals were fed irradiated NIH-07 pellets or wafers (Zeigler Bros., Gardners, PA). After weaning, animals were fed NTP-2000 (Zeigler Bros., Gardners, PA). Rats were provided municipal water *ad libitum* from an automatic watering system; both water and feed were analyzed for known contaminants that could interfere with or affect the outcome of the study, and none were found. Animals used in this investigation of the mucosa-associated microbiome were from a larger toxicology study that will be reported elsewhere (manuscript under preparation). The experimental design is outlined in Figure 1.

### HIV dose selection and exposure to animals

There were 3 groups in this study (n = 5 per group). Group 1 (control): 0 mg/kg/day dosed with vehicle; Group 2 (low dose): 150/12.5/75 mg/kg ABC/DTG/3TC; Group 3 (high dose): 300/25/150 mg/kg ABC/DTG/3TC. Vehicle was 0.2% methylcellulose/0.1% Tween 80. The treatment regimen and administered doses were based on our previously published study, which used the same animal cohort and provided detailed justification for dose selection [27]. In summary, the doses were chosen based on human therapeutic relevance and previously established toxicological data.

TC-ART was administered via oral gavage to time-mated female Sprague Dawley rats (Hsd:SD) starting on gestational day 6 (GD6) and continued through gestation, and lactation (PND21) (NTP study T06055). F1 offspring were never directly dosed in this study. Dams were weighed approximately every 3 days, and dosing volumes (5 mL/kg) were based on the most recent body weight. One male and one female offspring from each dam were used for the study.

### Collection of ileal tissue for DNA extraction

After the pups were weaned from the dams, they were housed up to five per sex per cage and provided food and water *ad libitum*. Once the offspring reached 12 months of age, which is comparable to approximately 26 years in humans [28], they were euthanized by exposure to carbon dioxide, strictly adhering to the guidelines set by the IACUC. In a sterile environment, the ileum was exposed and flushed with sterile PBS to remove feces. The samples were immediately placed in pre-labelled tubes, frozen in liquid nitrogen and transported overnight on dry ice to the National Center for Toxicological Research (NCTR), Jefferson, AR, for analysis.

### Ileal mucosa-associated microbial DNA extraction for population analysis

Maintaining the sterile conditions, a small section of the ileal tissue was placed in CTAB extraction solution and finely minced using a scalpel. The minced tissue was transferred into bead-beating tubes (lysing matrix B) and processed using a Fast prep Machine at 6 m/s for 45 s. Thus, obtained lysate was incubated with proteinase k (at 65°C for 20 min), followed by RNase-A (at 37°C for 15 min) as described earlier [29]. To this an equal volume of phenol-chloroform-isopropanol (PCI) solution was added and centrifuged at 12,000xg. The supernatant aqueous solution was separated, and the DNA was precipitated using sodium acetate, isopropanol, and a polyacryl carrier. The DNA pellet was washed with 70% ethanol and then dissolved in nuclease- free water. The DNA was quantified with a Qubit fluorometer (Thermo Fisher Scientific, Waltham, Massachusetts) and subsequently used for sequencing.

### Rat mucosa-associated microbial population amplicon sequencing

The samples were processed and standardized using a modified methodology based on Gohl et al. 2016 [30]. The KAPA HiFi Polymerase from KAPA Biosystems was used to amplify the variable region 4 (515f/806r) of the 16S rRNA gene. The libraries were subjected to sequencing on a MiSeq (Illumina) instrument, with paired-end 2 × 250 reads, using the MiSeq Reagent Kit V3 (Illumina, 600 cycle kit), aiming to achieve a target depth of 50,000 reads. The sequencing reads underwent adapter and primer removal using Cutadapt, and any reads with a Q score below 30 were discarded. The amplicon sequence variations (ASVs) were generated using dada2 v1.16.0, using quality-controlled reads. As part of the dada2 workflow, the paired FASTQ reads underwent trimming and filtering to eliminate reads that had ambiguous nucleotides (represented by “N”s) or more than 2 sequencing mistakes per read. The dada2 learn error rate model was utilized to estimate the error profile prior to employing the core dada2 algorithm for inferring the sample composition. The forward and reverse readings were combined, and any chimeras were eliminated prior to taxonomy classification. The ASV taxonomy was determined up to the level of species using the SILVA v.138 database, following the technique developed earlier [31]. A minimum bootstrapping support of 50% was required for taxonomic assignment. Species-level taxonomy was awarded to ASVs based only on 100% identity and unambiguous reference matching.

### Statistical/data analysis

Microbiome data was visualized using either microbiome analyst [32] or metaboanalyst websites [33]. For the microbiome analysis, the software used an ANOVA, followed by *post hoc* pairwise comparisons using Mann-Whitney and Kruskal-Wallis statistical tests. For the comparison between two groups either EdgeR or student T-tests were conducted. The samples for the microbiome analyst were normalized to remove 19 low abundance and 11 low variance values based on mean and standard deviation respectively. While for the metaboanalyst the samples normalization was performed using the normalization by sum. Following that the data scaling was applied using mean centering and log transformation options to ensure approximate normal distribution.

## Results

Comparative analysis of the treatment groups (n = 5 per group) was conducted to assess the impact of gestational and lactational exposure of TC-ART on microbial profiles, focusing on sex-specific impacts on the offspring. During the analysis one control female and one high dose female animal showed deviation in the microbial profile compared to the other animals in respective experimental groups. However, the outlier test showed the two animals are not statistically classified outliers. We have excluded the animals for the alpha diversity but to maintain the integrity, we have included the animals in our dataset for the rest of the analysis. The inclusion of these animals for later analysis did not alter our conclusions, rather it highlights the natural variability within the controlled experimental groups.

### Sex-specific microbiota differences and differential response to exposure are observed in male and female animals

**FIGURE 2:**
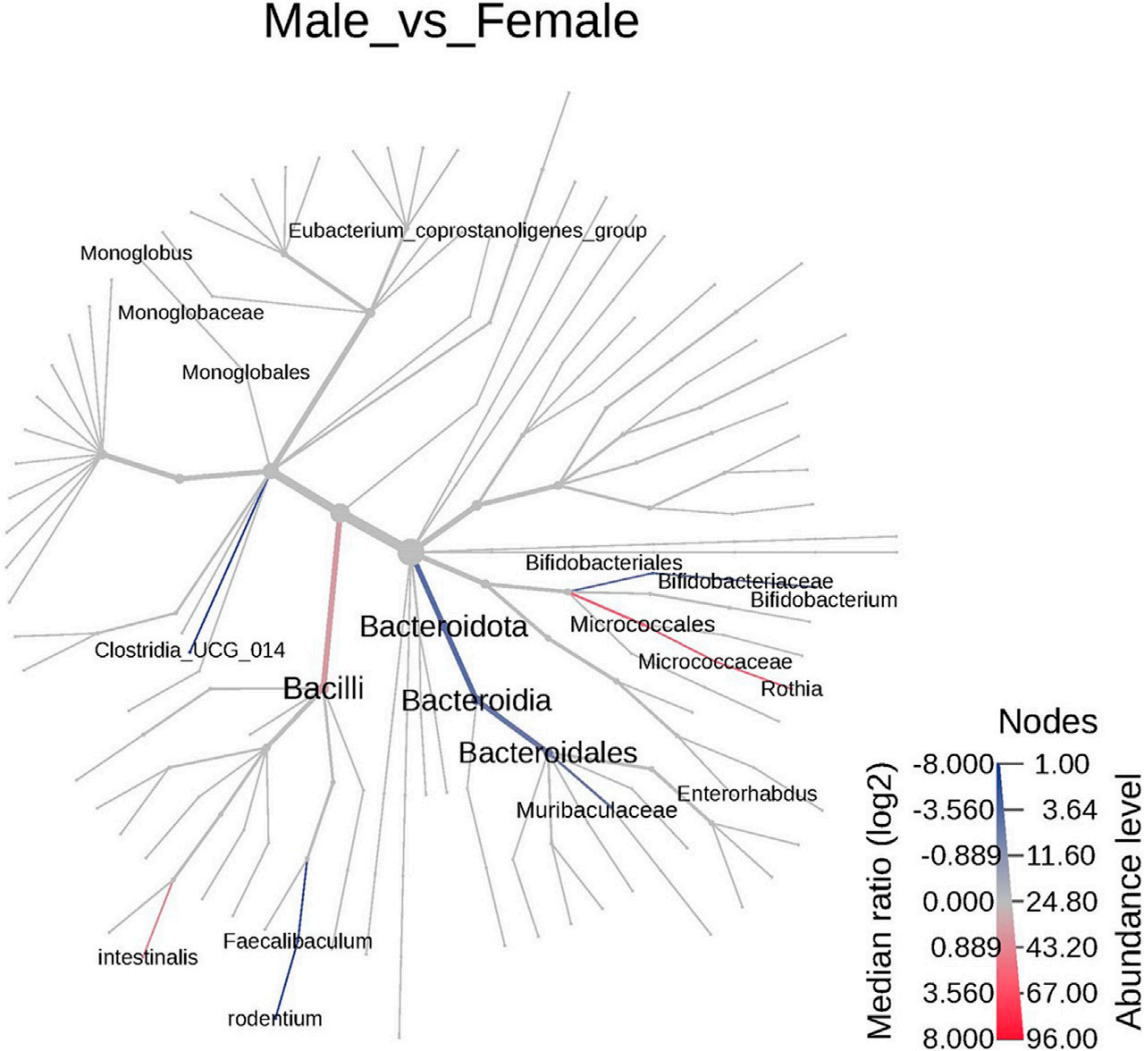
The cladogram represents the phylogenetic relationship between the male and the female animals between different taxa. The blue or highlighted branches represent significant decreased taxa in females while the red represent the significantly increased taxa. The branches are colored based on median log2 ratios and the intensity of color represents the abundance. Significant differences were observed in *Bacteroidales* and *Bacili*.

Sex-specific differences were observed between the microbiota of the control animals. A total of 37 significant features were differentiated between the female and male animals (Supplementary Table S1). At the phyla and species level 2 significant features were identified, 3 at family level, and 32 features varied at the genus level. The cladogram analysis revealed notable differences between the male and the female animals at the genus level (Figure 2). Notably the *Bacteroidota* and *Bifidobacteriales* were significantly increased in the female animals compared to the male animals while the *Clostridia, Bacilli,* and *Eubacterium coprostanoligenes group* were decreased in the female animals. The male and the female animals have responded differently to TC-ART exposure. While the exposure in male animals resulted in increase of opportunistic bacteria the female animals showed decrease in the abundance of opportunistic bacterial species. To have a more granular view on the differences we analyzed the changes in the microbiome upon exposure at different taxonomic levels.

### Mucosa associated microbiome diversity changes with 16s amplicon sequencing data

The ileal mucosa-associated bacterial population in the adult offspring perinatally exposed to TC-ART was assessed by 16s amplicon sequencing.

**FIGURE 3:**
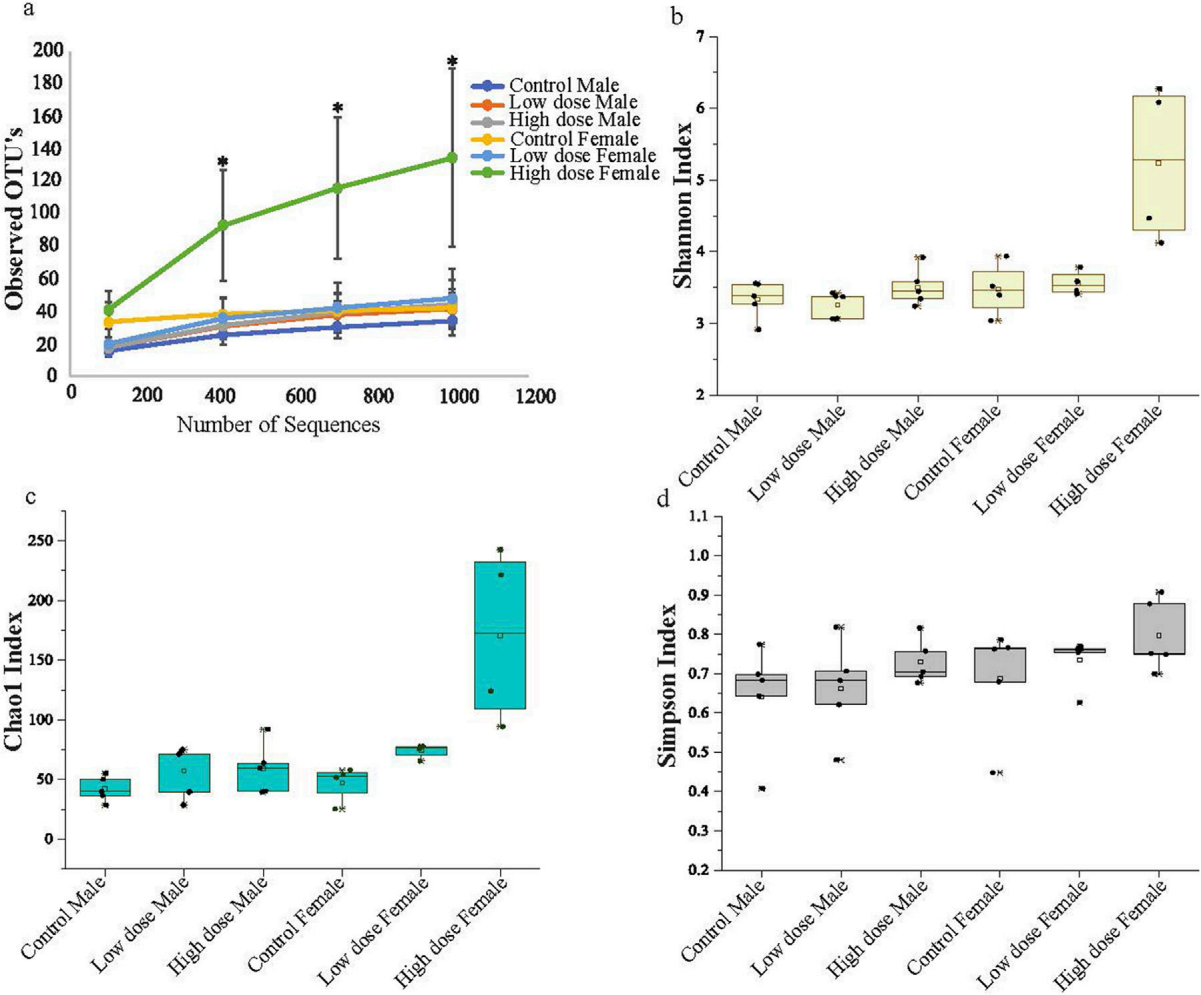
Alpha diversity **(a)** Observed OTU abundance over sequence number. The figure shows the observed operational taxonomic unit (OTU) abundance as a function of sequence number for male and female animals across three experimental groups: control, low dose, and high dose treatment. Each line represents the mean OTU abundance for each group. The high dose exposed female animals display a higher OTU abundance compared to the other groups. **(b)** Shannon Diversity Index. **(c)** Chao1 Index **(d)** Simpson Diversity Index. Illustrates the diversity of microbial communities in male and female animals across the groups. The high dose treated female animals show significant differentiation from the other groups, indicating a notable shift in microbial diversity and richness associated with higher treatment levels. The data represents mean with standard deviation (n = 5; *p < 0.05).

The changes in bacterial diversity, richness, and evenness in each group were evaluated by using different alpha diversity indices including total number of observed operational taxonomic units (OTUs), Chao1, Shannon, and Simpson as shown in Figure 3. The female high dose offspring exhibited significantly higher species richness compared to all other groups, with an observed OTU of 132 (Figure 3a); results that were further confirmed when evaluating the Shannon and Chao1 diversity indices (Figures 3b, c). Interestingly, female control animals showed higher species richness compared to male animals, indicating base line sex differences. The microbial species richness in the high dose exposed female offspring were significantly higher compared to other groups (Figures 3a–c; Supplementary Table S2), suggesting that maternal administration of the drugs at the high dose resulted in the enhanced species richness and rare species (low abundant species) in these animals. Conversely, no significant differences between groups were observed using the Simpson Index, which measures richness and evenness between bacterial communities. This suggests a similar level of species dominance within bacterial community among all tested animals (Figure 3d). The rarefaction curves also suggest higher species richness at higher sequence depths in both male and female animals in the high dose-exposed groups (Supplementary Figure S1).

### Perinatal exposure to TC-ART leads to taxonomic level changes at phyla, genus, and species levels

**FIGURE 4:**
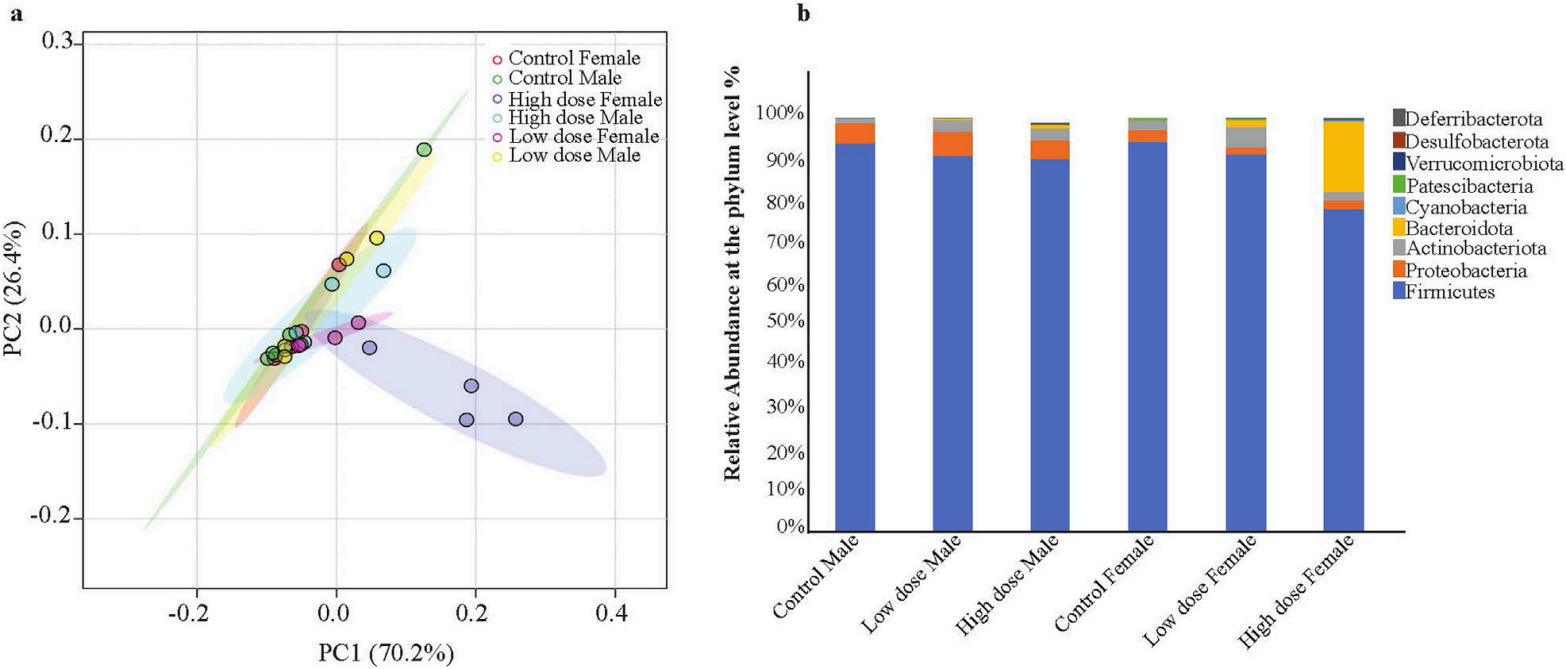
Principal Component Analysis (PCA) of microbiome profiles and changes in gut microbiota composition in male and female rat pups exposed to Tricombo drug. **(a)** PCA of the microbial profiles. The PCA score plot illustrates the clustering of microbiome profiles from each group. The plot shows ellipses indicating the 95% confidence intervals with the high dose female (blue) distinctly separated from the other groups. **(b)** Relative abundance of gut microbiota at the phyla level: The bar diagram represents the relative abundance of different bacterial phyla in male and female rat pups exposed to low and high doses of the tricombo drug compared to control animals. The bacterial phyla include Firmicutes, Proteobacteria, Actinobacteriota, Bacteroidota, Cyanobacteria, Patescibacteria, Verrucomicrobiota, Desulfobacterota, and Deferribacterota. The High dose exposed female animals has shown significant variations in *Firmicutes/Bacteroidota* ratio compared to other groups. Data is represented as the percentage of total bacterial abundance for each group Control Male, Low dose Male, High dose Male, Control Female, Low dose Female, High dose Female.

The principal component analysis (PCA) at the phylum level showed a distinct shift in the clustering of the high dose exposed female animals (Figure 4a), correlating with significant alterations in Firmicutes/Bacteroidetes ratio (Figure 4b). The low dose showed a substantial increase of Bacteroidetes relative microbial abundance compared to the control animals, while the high dose showed an even greater increase. The Bacteroidetes change was consistent across all the female high dose exposed animals (Figure 4b). The relative abundance of Firmicutes was reduced with the treatment. Although the males also showed a similar trend, the variations were not as prominent as in female animals (Figure 4b; Supplementary Figure S2). Treated male animals had increased Proteobacteria phyla compared to the control animals, while females had a decrease (Figure 4b). Similarly, an increase in the phyla Deferribacterota was observed in the high dose exposed female rats relative to the control animals. Of note, males from the high dose-only and females from both dosed-groups have more unidentified phyla, indicating a diversified bacterial population associated with drug exposure. This, in turn, could potentially lead to metabolic disturbances.

**FIGURE 5:**
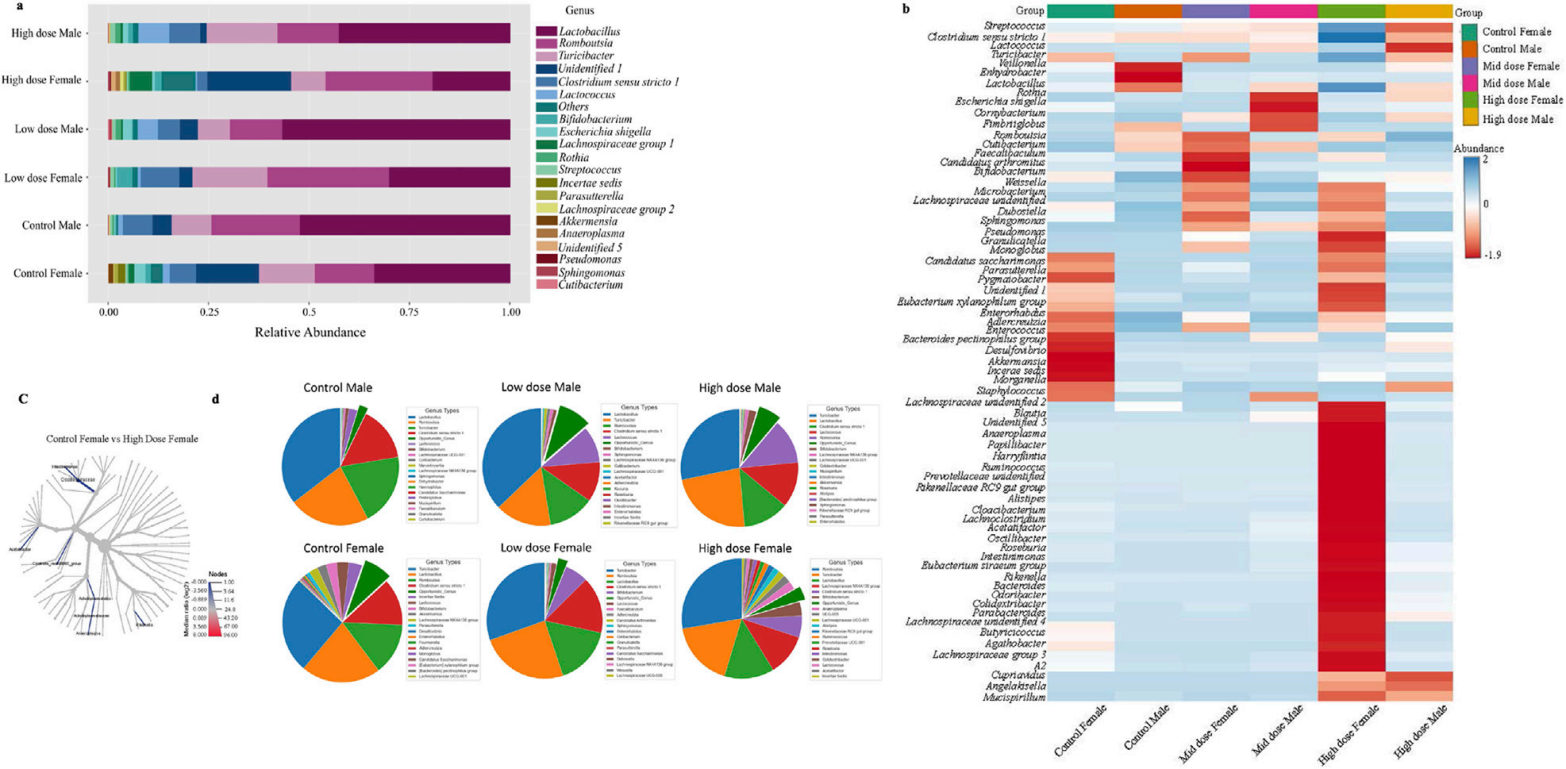
Relative abundance of bacterial genus in different TC-ART exposure groups. **(a)** The stacked bar plot illustrates the composition of gut microbiota in male and female subjects across groups: Control Male, Low dose Male, High dose Male, Control Female, Low dose Female, and High dose Female. Each color in the bars represents a different bacterial genus. *Lactobacillus* (yellow), *Romboutsia* (green), and *Turicibacter* (reddish pink), *Lactococcus* (orange), *Clostridium sensu stricto 1* (grey) show significant alterations. High dose treatments decreased *Lactobacillus* and Lactococcus with more pronounced changes in females. These variations highlight the impact of exposure on gut microbiota composition. **(b)** Heatmap of bacterial genera distribution across different exposure doses and in control animals. The rows correspond to bacterial genera, and the columns to the samples within each group. The color scale indicates the abundance of each genus, ranging from blue (low abundance) to red (high abundance). A distinct microbial profile observed in the high dose exposed female animals compared to other groups, indicating a significant shift in bacterial genera due to the exposure. **(c)** The cladogram represents comparison between the control female and the high dose female animals. The blue highlighted branches represent significant taxa and the taxa with higher median log2 ratios in high dose female group are more abundant compared to the control animals indicated by color gradient and intensity. **(d)** The pie chart of top 20 abundance genera with the opportunistic bacterial genera highlighted between the groups (dark green). The abundance of the opportunistic bacteria was added and represented in the pie diagram. The exposed male animals have shown increased abundance of opportunistic bacterial genera while the females have shown low abundance with the exposure to TC-ART.

The identified changes in bacterial diversity described above might differentially influence downstream effects. To determine if this was the case, we next analyzed bacteria composition at the genus and species level, which can provide information on their pathogenic or commensal origins. We observed notable sex- and treatment- dependent variabilities. The major genera showed comparable differences between the male and female control animals (Figures 5a, b). Beneficial genera such as *Lactobacillus, Lachnospiraceae, Lactococcus, Romboutsia, and Bifidobacterium,* that promote gut health showed dose-dependent alterations (Figures 5a, b). Interestingly, *Lactobacillus* genera has shown a dose dependent decrease in all groups while *Romboutsia* from Firmicutes phyla showed a decrease in male animals (Figure 5a). Conversely, the genera associated with inflammation or pathogenicity, including *Escherichia (Escherichia schigella), Pseudomonas, Angelakisella,* and *Parabacteroides,* were increased in the groups exposed to TC-ART. Notably, *Pseudomonas*, which is linked to gut dysbiosis and immune activation, showed a dose-dependent increase in both male and female subjects. *Clostridium* sensu stricto1 was decreased in females in both exposed groups but not changed in male groups (Figures 5a, b; Supplementary Figure S3a).

Genera such as *Cupriavidus, Odoribacter*, and *Tyzzerella* were uniquely present in both male and female high dose exposed groups. The abundance of some genera like *Bacteroides, Butyricoccus, Turicibacter,* and *Eubacterium (Eubacterium ventriosum, Eubacterium siraeum)* showed transient changes in response to treatment indicating sensitivity of the bacterial community following prenatal exposure to the ART (Figure 5b).

When single-factor statistical comparisons were performed using EdgeR, a total of 19 significant features were altered at the genus level between the control female and high dose female animals using PCA analysis (Supplementary Figure S3b). Additionally, 15 microbial features differed, including 9 variations at the genus level, were identified between the control male and the female animals, consistent with the notion of base line sex-specific differences described above. The cladogram (Figure 5c) depicts significant alterations in the relative abundance of several taxa such as *Oscillospiraceae, Intestinimonas, Acettatifactor, Rikenella, Anaeroplasma, Acholeplasmatales*, and *Clostridia VadinBB60* group, are significantly enriched in high dose female group. The shift in Oscillospiraceae and Intestinimonas families in the high dose-exposed animals was notable. Also, females showed higher abundance of *Bifidobacterium* while males showed increased levels of *Clostridia*. Changes were observed in genera within the *Bacteroidetes* and *Bacilli* with more opportunistic genera in the control female animals compared to the male animals, which were further changed in the exposed groups (Figure 5d).

**FIGURE 6:**
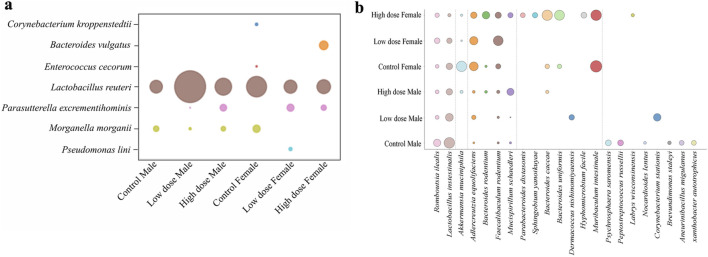
Comparative analysis of mucosa associated microbial species across different groups. Bubble plots representing the relative abundance of **(a)** opportunistic bacterial species in mucosa tissue **(b)** commensal bacterial species across the treatment groups in mucosa tissue.

To have a more granular view at the taxonomy, we investigated abundance of bacterial species in each experimental group, which revealed dose- and sex-dependent changes (Figure 6a). Protective bacteria like *Lactobacillus intestinalis and Akkermansia muciniphila* decreased while other commensal bacteria *Bacteroides uniformis, Bacteroides caccae,* and *Mucispirillum schaedleri* were increased in treatment groups (Figures 6a, b). Interestingly, most of the commensal bacteria showed lower abundance in the low dose exposed animals compared to the high dose exposed animals.

## Discussion

The ability of TC-ART to cross the placental barrier and its presence in milk invite a better understanding of the risks associated with their use in pregnancy and lactation [34]. Numerous studies have explored the effects of TC-ART on the mother and the offspring, focusing on aspects like cardiovascular diseases, neuronal development, immune health [35]. These studies focus on the effects of the drugs on these tissues, but often neglect to include analysis on the effects on the gut health of the offspring. Given the importance of gut health and the microbiome in maintaining individual homeostasis, and their association with several pathological conditions when disrupted, it is important to consider how they may be affected by early drug exposure. Perinatal TC-ART exposure may alter the gut microbiome that could impact metabolism, intestinal permeability, and immune system contributing to long-term health outcomes. In this study, by investigating mucosa-associated microbial changes in rats that were exposed to TC-ART solely through placental and lactational transfer, we found significant changes in the composition of intestinal microbiome in adulthood.

Our data aligns with the understanding that the fecal microbiome is more abundant and diverse than the mucosa-associated microbiome. Although both fecal and mucosal microbiomes showed differences, the changes were more pronounced in the mucosal microbiome. This may suggest that the mucosal environment is more sensitive to high-dose exposure and exhibits differential responses, possibly due to the drug being primarily absorbed in the small intestine [27, 36]. Furthermore, several studies have shown that the mucosal environment is crucial for microbiome-immune interactions due to its proximity with the intestinal epithelium and pattern recognition receptors (PRRs) [37, 38]. The combined effect of mucosal environment along with the presence of TC-ART could have significantly contributed to observed shifts in mucosal microbiome. Interestingly, the mucosal data showing a higher proportion of opportunistic bacteria and decreased commensal bacteria, along with the differences in the fecal and mucosal microbiome of the control animals, highlights the importance of studying both to understand the dynamics of drug exposure.

Overall, our data showed sex-dependent and dose-dependent drug-induced changes in the gut microbiome, which were more pronounced in females than males. Notably, sex-dependent microbial variations between male and female control animals were identified, which may provide insights for understanding the sex-specific microbial functions or outcomes. Although female animals exhibited lower microbial abundance, their higher observed OTU counts compared to male samples suggests greater microbial diversity in females. Additionally, treatment in both male and females led to reduced microbial abundance indicating a non-favorable effect of the exposure on the gut microbiome. The opportunistic genera in the male animals increased with the exposure while it decreased in the female animals, suggesting sex-based differences in the effects of the drugs. Sex hormones such as estrogen and testosterone are known to influence gut microbial composition, diversity, and functions [39]. In healthy women, higher estrogen levels are associated with increased Bacteroidetes and microbial diversity, while in healthy men, elevated testosterone levels correlate with higher levels of *Ruminococcus* and *Acinetobacter*. However, in women, higher testosterone levels are associated with *Escherichia* and *Shigella* species, while *Ruminococcus* species; is negatively associated with elevated testosterone levels [40]. The increased Bacteroidetes and reduced Firmicutes ratio in high dose females observed in our study is often associated with several pathologies including IBS and immune profile alterations [41]. Generally, decreases in Firmicutes and increases in Bacteriodata are considered ideal but high changes in the composition might also disrupt the digestive function and other metabolic pathways. Some studies have revealed that similar microbial changes observed in female animals are associated with disease outcomes in patients with Alzheimer’s, refractory epilepsy, and some neurological diseases [42–44]. Alterations in *Actinobacteria* and *Proteobacteria* in the treated groups suggest microbial dysbiosis, which has been linked to inflammatory diseases [41], coronary heart failure, and other cardiac abnormalities [45–47]. For example, *Romboutsia ilealis* species is often linked with better gut health, including improved metabolism, lowering dietary inflammation, and controlling blood sugar and fat [48, 49]. The enrichment of *Romboutsia* in the female animals could contribute to decreased proinflammatory cytokines production. Similarly, the changes in abundance of the *Clostridium sensu stricto1, Escherichia, shigella,* and *Enterococcus* observed in the treated groups have been associated with metabolic dysfunction and fatty acid liver disease [50, 51], as well as heart failure and coronary artery diseases [47]. [52]. Notably, these animals do show cardiac dysfunction later in life (Taube et al., in preparation), but whether the changes in the microbiome reported here play a role in that phenotype remain unclear.

The reduced abundance of *Akkermansia mucinophila* is often associated with vascular inflammation, endotoxemia, and barrier dysfunction [45, 53]. *Akkermansia mucinophila* is a mucin degrading bacterial community that modulates expression of tight junction genes like occludins [53]; thus can influence the intestinal permeability and induce translocation of pathogenic bacteria into the systemic circulation that could contribute the metabolic diseases. Another species, *Faecalibaculum rodentium,* known for its cholesterol degrading activity and protective effects against intestinal tumor growth, was slightly increased with the treatment in both male and female animals. This species is also known to induce oxidative stress and inflammation [54, 55]. The differential abundance of *Aslercreutzia eqyolifaciens* species is related to several disease conditions such as metabolic liver diseases [56]. Given that most of the bacteria are initially acquired from the mother and the surrounding environment, the coprophagy during the co-housing of dam and pups during early development could be another possibility of the transfer of altered microbial population (due to impact of drug on the intestinal microbiota) from dams to pups [57, 58]. These microbial changes may persist into adulthood influencing the microbiome composition of the offspring.

In conclusion, our study shows the effects of TC-ART on the mucosal microbiome of the adult offspring of animals exposed through gestation and lactation. The differences observed highlight the potential sensitivity of the mucosal environment to the drug exposure. The extent to which these changes might affect other phenotypes on the animals, including impacts on their cardiovascular health, is unclear but deserves further investigation. Since our data also suggest sex-specific differences, further studies on the influence of sex hormones to the phenotypes here described are also warranted.

## Data Availability

The datasets presented in this article are not readily available because the work is conducted at the government institute. Requests to access the datasets should be directed to the corresponding author.
