# Supplementary material for: Gestational exposure to HIV drugs alters intestinal mucosa-associated microbial diversity in adult rat offspring
Source: Exp Biol Med (Maywood). 2025 Aug 13;250:10564. doi: 10.3389/ebm.2025.10564 (PMC12382514; doi:10.3389/ebm.2025.10564)
Supplement: Supplementary file 1 [file Supplementaryfile1.docx]

Supplementary data:

S1


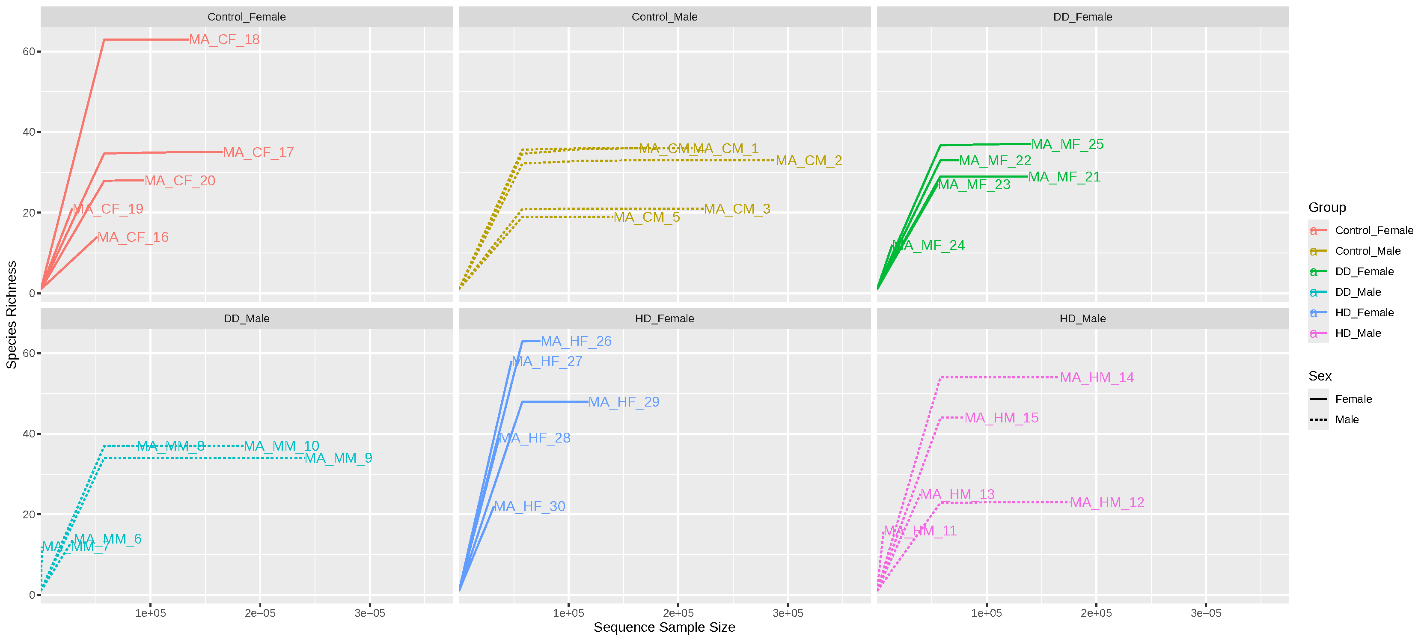


Supplementary Figure S1: Rare fraction curves in different treatment groups control, DD (Low dose), HD (high dose).

S2


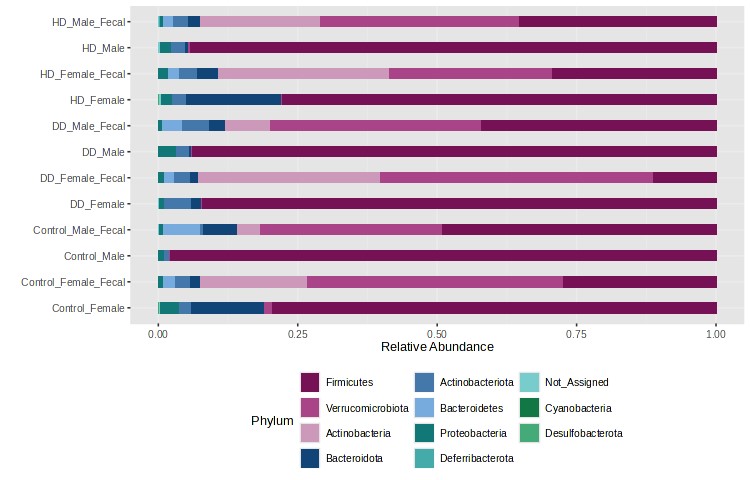


Supplementary Figure S2: Abundance of the male and female animals at the phyla level in the fecal samples in groups Control Female, Control Male, Low dose Female (DD_Male), Low dose Male (DD_Female), High dose Female (HD_Female), High dose Male (HD_Male).

S3

a


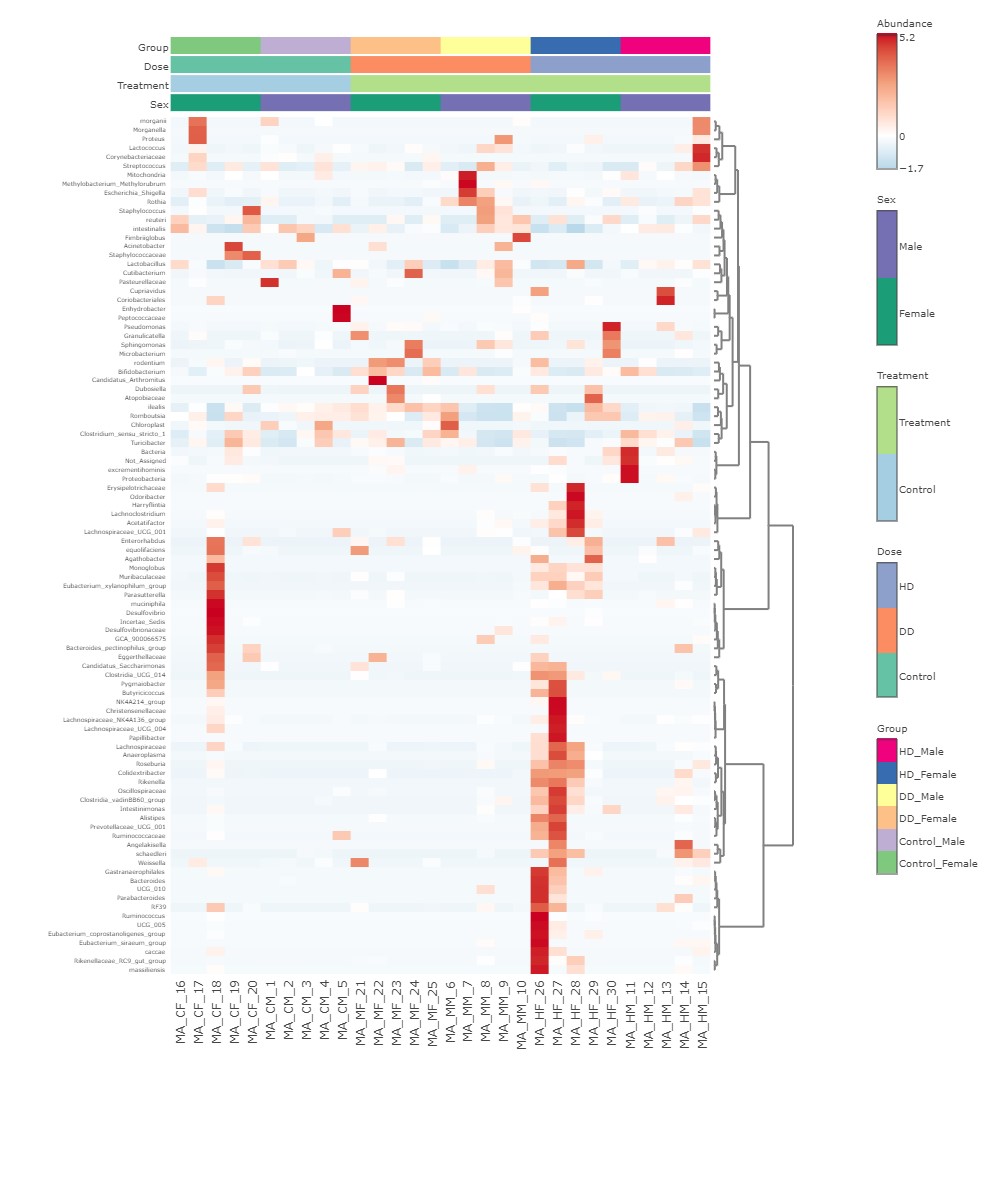


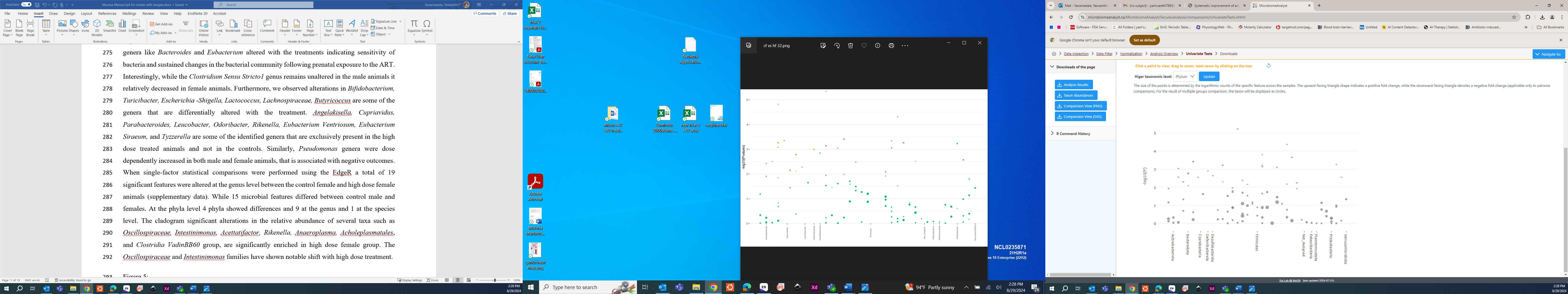


b

Supplementary Figure S3: a) Relative abundance of bacterial genus for each treatment groups in individual samples (MA represents mucosa, DD represents low dose, and CF-control female, CM-control male, MF-Low dose female, MM-Low dose male, HF-high dose female, HM-high dose male) b) Single-factor statistical comparision between control female and high dose female showing 32 significant differences with 19 significant variations at the genus level. The size of the bubble represents the abundance the upward triangle represents the upregulation, and the downward triangle represents downregulation.

Supplementary Table 1: EdgeR statistical comparision between male and female groups

|  | log2FC | logCPM | Pvalues | FDR |
| --- | --- | --- | --- | --- |
| *Staphylococcaceae* | -6.234 | 9.4459 | 1.14E-06 | 4.25E-05 |
| *Monoglobus* | -4.6092 | 7.9152 | 1.15E-06 | 4.25E-05 |
| *Eubacterium_coprostanoligenes_group* | -4.5787 | 7.8874 | 1.33E-06 | 4.25E-05 |
| *Prevotellaceae_UCG_001* | -5.3314 | 8.8386 | 1.90E-06 | 4.57E-05 |
| *Enhydrobacter* | 5.0088 | 8.1068 | 6.78E-06 | 0.000124 |
| *NK4A214_group* | -4.5195 | 7.8378 | 7.74E-06 | 0.000124 |
| *Eggerthellaceae* | -3.6306 | 7.0643 | 1.37E-05 | 0.000187 |
| *Muribaculum_intestinale* | -4.0747 | 7.4476 | 1.88E-05 | 0.000225 |
| *Clostridia_UCG_014* | -4.4336 | 11.667 | 3.50E-05 | 0.00032 |
| *Gastranaerophilales* | -3.3333 | 7.2342 | 3.79E-05 | 0.00032 |
| *Haemophilus* | 3.8788 | 7.1032 | 3.83E-05 | 0.00032 |
| *Harryflintia* | -4.039 | 7.4152 | 4.00E-05 | 0.00032 |
| *Fimbriiglobus* | 3.5892 | 6.8609 | 7.26E-05 | 0.000536 |
| *Atopobiaceae* | -3.2424 | 6.7578 | 0.000134 | 0.000906 |
| *Ruminococcus* | -2.9621 | 6.8599 | 0.000142 | 0.000906 |
| *Bacteroides_uniformis* | -3.0882 | 6.638 | 0.000175 | 0.001048 |
| *Cloacibacterium* | -3.5737 | 7.4152 | 0.000198 | 0.001121 |
| *Muribaculaceae* | -3.9085 | 14.657 | 0.000298 | 0.001587 |
| *Eubacterium_xylanophilum_group* | -3.0221 | 7.6418 | 0.000371 | 0.001872 |
| *Anaeroplasma* | -4.0941 | 10.49 | 0.000685 | 0.003289 |
| *Christensenellaceae* | -2.6468 | 6.316 | 0.000998 | 0.004561 |
| *Pygmaiobacter* | -2.5484 | 6.8024 | 0.001159 | 0.005059 |
| *Peptococcaceae* | 3.4167 | 7.9362 | 0.001245 | 0.005195 |
| *Incertae_Sedis* | -3.6158 | 10.603 | 0.001431 | 0.005722 |
| *Lachnospiraceae_UCG_006* | -2.6903 | 7.8772 | 0.002902 | 0.011112 |
| *Alistipes* | -3.2171 | 9.3555 | 0.00301 | 0.011112 |
| *Cupriavidus* | 2.1659 | 6.1535 | 0.003517 | 0.012505 |
| *Butyricicoccus* | -2.1283 | 5.9621 | 0.003649 | 0.01251 |
| *Parasutterella* | -3.1945 | 10.575 | 0.005499 | 0.018203 |
| *Dubosiella* | -2.5554 | 8.1039 | 0.007572 | 0.024229 |
| *Odoribacter* | -2.4888 | 7.4377 | 0.008728 | 0.027029 |
| *Methylobacterium_Methylorubrum* | 2.0204 | 6.3856 | 0.009967 | 0.028792 |
| *Cutibacterium* | 3.0387 | 11.226 | 0.010197 | 0.028792 |
| *Veillonella* | 2.4452 | 7.897 | 0.011603 | 0.030941 |
| *Erysipelotrichaceae* | -1.9071 | 6.3738 | 0.012502 | 0.032438 |
| *Pasteurellaceae* | 3.4317 | 15.05 | 0.018197 | 0.04597 |
| *Lactobacillus* | 1.685 | 18.522 | 0.020126 | 0.049542 |
| *Faecalibaculum_rodentium* | -2.3623 | 9.9065 | 0.021818 | 0.052363 |
| *Lactococcus* | 2.3242 | 15.325 | 0.022862 | 0.053531 |
| *Weissella* | -1.999 | 7.8563 | 0.024294 | 0.054345 |
| *Agathobacter* | -1.741 | 6.4173 | 0.024342 | 0.054345 |
| *Akkermansia_muciniphila* | -2.5189 | 10.002 | 0.024908 | 0.054345 |
| *UCG_005* | -2.1201 | 8.0246 | 0.02691 | 0.057407 |
| *Bacteroides_massiliensis* | -1.5744 | 6.6136 | 0.0311 | 0.064905 |
| *Blautia* | 1.5235 | 6.2029 | 0.036728 | 0.07502 |
| *Colidextribacter* | -2.0141 | 9.1989 | 0.041179 | 0.082357 |
| *Rothia* | 1.7483 | 13.28 | 0.047312 | 0.092692 |
| *Enterorhabdus* | -2.1062 | 9.6276 | 0.048409 | 0.092945 |
| *Lactobacillus_intestinalis* | 1.4231 | 18.46 | 0.049399 | 0.092986 |

Supplementary Table 2: EdgeR statistical comparision between control and high dose female treatment groups

|  | log2FC | logCPM | Pvalues | FDR |
| --- | --- | --- | --- | --- |
| *Staphylococcaceae* | 8.4378 | 9.4456 | 6.01E-06 | 0.000577 |
| *Harryflintia* | -5.8431 | 7.4154 | 4.32E-05 | 0.002071 |
| *Bacteroides_pectinophilus_group* | 5.532 | 7.128 | 0.00016 | 0.005111 |
| *Anaeroplasma* | -6.8679 | 10.49 | 0.000225 | 0.005398 |
| *Prevotellaceae_UCG_001* | -5.3874 | 8.8395 | 0.000375 | 0.005921 |
| *Odoribacter* | -5.696 | 7.4383 | 0.00039 | 0.005921 |
| *Morganella* | 5.9214 | 7.5392 | 0.000555 | 0.005921 |
| *Staphylococcus* | 7.5884 | 9.7835 | 0.000608 | 0.005921 |
| *Eubacterium_coprostanoligenes_group* | -4.1563 | 7.8901 | 0.000615 | 0.005921 |
| *Oscillibacter* | -5.8999 | 8.0092 | 0.000617 | 0.005921 |
| *Clostridia_vadinBB60_group* | -5.9502 | 9.3565 | 0.000733 | 0.006398 |
| *Enterococcus* | 7.558 | 9.3864 | 0.000806 | 0.006447 |
| *Eggerthellaceae* | 4.1921 | 7.0662 | 0.000934 | 0.006894 |
| *Mucispirillum_schaedleri* | -5.3275 | 8.1552 | 0.001282 | 0.008793 |
| *Bacteroides* | -4.4104 | 6.8836 | 0.001645 | 0.010531 |
| *Rikenellaceae_RC9_gut_group* | -5.3326 | 8.6727 | 0.001795 | 0.010771 |
| *Desulfovibrionaceae* | 4.8103 | 7.264 | 0.002328 | 0.013145 |
| *Atopobiaceae* | -4.2851 | 6.7592 | 0.002763 | 0.013703 |
| *Morganella_morganii* | 5.778 | 7.9703 | 0.002815 | 0.013703 |
| *Gastranaerophilales* | -3.1811 | 7.2374 | 0.002855 | 0.013703 |
| *UCG_005* | -4.0244 | 8.0276 | 0.004219 | 0.019288 |
| *NK4A214_group* | -3.7827 | 7.8393 | 0.005013 | 0.021333 |
| *Alistipes* | -5.2264 | 9.3561 | 0.005111 | 0.021333 |
| *Oscillospiraceae* | -5.641 | 10.164 | 0.005787 | 0.023146 |
| *GCA_900066575* | 3.8962 | 6.9919 | 0.006764 | 0.025947 |
| *UCG_010* | -3.5831 | 6.5095 | 0.007027 | 0.025947 |
| *Bacteroides_uniformis* | -2.8856 | 6.6388 | 0.008219 | 0.029221 |
| *Intestinimonas* | -4.1087 | 9.5569 | 0.010803 | 0.037038 |
| *Corynebacteriaceae* | 4.1303 | 7.2348 | 0.012043 | 0.038635 |
| *Veillonella* | 3.9392 | 7.8978 | 0.012073 | 0.038635 |
| *Christensenellaceae* | -2.6667 | 6.3182 | 0.01516 | 0.046946 |
| *Ruminococcaceae* | -3.8254 | 8.8802 | 0.018048 | 0.054144 |
| *Acetatifactor* | -3.8975 | 9.5808 | 0.018712 | 0.054435 |
| *Colidextribacter* | -3.7566 | 9.1997 | 0.020311 | 0.057349 |
| *Ruminococcus* | -2.2945 | 6.8681 | 0.022797 | 0.062529 |
| *Angelakisella* | -2.8206 | 6.0026 | 0.029611 | 0.076829 |
| *Roseburia* | -3.6837 | 9.6125 | 0.030655 | 0.077443 |
| *Lachnospiraceae_UCG_001* | -4.7324 | 11.995 | 0.035767 | 0.088043 |
| *Akkermansia_muciniphila* | 3.6132 | 10.003 | 0.046383 | 0.11132 |
| *Bacteroides_massiliensis* | -2.1185 | 6.6189 | 0.047635 | 0.11153 |
